# Primary Cervical Extraosseous Ewing's Sarcoma Originated from the Sternocleidomastoid Muscle: A Case Report and Review of the Literature

**DOI:** 10.1155/2024/8867131

**Published:** 2024-02-22

**Authors:** Sasa Jakovljevic, Nenad Arsovic, Zoran Dudvarski, Nemanja Radivojevic, Katarina Jovanovic, Neda Mladenovic, Snezana Babac

**Affiliations:** ^1^University of Belgrade, Faculty of Medicine, Belgrade, Serbia; ^2^University Clinical Center of Serbia, Clinic of Otorhinolaryngology and Maxillofacial Surgery, Belgrade, Serbia; ^3^Clinical Hospital Center Zvezdara, Belgrade, Serbia

## Abstract

Extraosseous Ewing's sarcoma is extremely rare in the soft tissues of the neck, especially in the sternocleidomastoid muscle. It usually manifests clinically as a rapidly growing mass that shows great potential for local spread. The aim of this paper is to present a rare case of еxtraosseous Ewing's sarcoma in the sternocleidomastoid muscle. To the best of our knowledge, this is the first case of extraskeletal Ewing's sarcoma at this location. The patient was admitted to our clinic because of a neck tumefaction. The computerized tomography finding showed a tumor mass, most of which was in the V region of the neck, measuring 40 × 27 × 35 mm. Pathohistological and immunohistochemical findings showed that it was Ewing's sarcoma. Unfortunately, the patient passed away nine months after the initial diagnosis. Extraosseous Ewing sarcoma is a rare, fast-growing malignant tumor manifesting histomorphological similarities to bone Ewing's sarcoma. Most reports state that extraosseous Ewing sarcoma has a worse prognosis than skeletal. Extraosseous Ewing sarcoma should be borne in mind in the differential diagnosis of soft tissue tumors of the neck.

## 1. Introduction

Ewing sarcoma family of tumors include a well-defined group of neoplasms that includes Ewing's sarcoma of the bone, extraosseous Ewing's sarcoma (EES), Askin tumor, and malignant primitive neuroectodermal tumor [1–4]. They are histologically similar, small, blue, and round tumors, all of which show a characteristic 11q22q chromosomal translocation [1]. Ewing sarcoma that occurs in the bone is the most common tumor in this family. Tumors that start in soft tissues but they look very much like Ewing sarcomas in bones are also known as extraskeletal Ewing sarcomas. Peripheral primitive neuroectodermal tumor that start in the chest wall are known as Askin tumor. These tumors are thought to typify a continuum of disease based on the degree of neuroectodermal differentiation [4]. EES was first described by Tefft et al. in 1969, and it is rare compared to Ewing's sarcoma in the bone [1, 5, 6]. Most cases reported in the literature are localized in the lower extremities and paravertebral region, and only a few are in the soft tissues of the neck. To the best of our knowledge, this is the first case of EES in the sternocleidomastoid muscle.

The frequency of EES in the head and neck region reported in the literature varies widely. So far, this form of ES has been described in the nose, orbit, larynx, nasopharynx, parotid, and submandibular glands [7]. A spectrum of immunohistochemical markers is used to study EES. These markers include CD99 antigen, which is a highly sensitive but not specific, and FLI1, which has higher specificity than CD99 [5]. Molecular genetic analysis include fluorescence in-situ hybridization that is essential. Clinically, it usually manifests as a rapidly growing mass with great potential for local spreading. Finally, it is an aggressive tumor with a high rate of local recurrences and distant metastases, and poor prognosis [8]. According to the NCCN and the European Society for Medical Oncology, all members of the Ewing family can be treated with the same protocol for localized disease that is local treatment in addition to chemotherapy.

This study aimed to present a rare case of EES in the sternocleidomastoid muscle in a 56-year-old man.

## 2. Case Report

**Figure 1:**
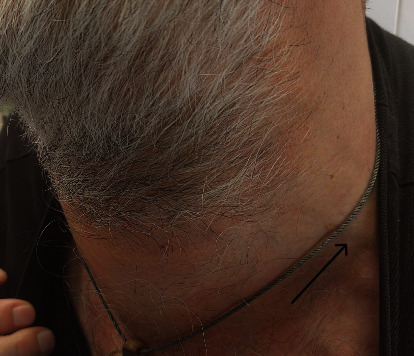
Photograph of the patient's neck-the arrow indicates the tumor mass in the IV and V regions of the neck.

**Figure 2:**
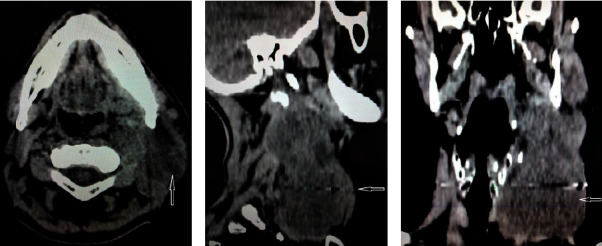
Computerized tomography of the neck shows a large tumor mass on the left side. (a) Axial view (b) sagittal view (c) coronal view.

A 56-year-old patient was admitted to our clinic because of a tumefaction on his left side of his neck, which he noticed 6 months earlier and was gradually increasing (Figure 1). Clinical examination revealed a tumor in the neck, about 3 cm in size, while the rest of the ENT findings were normal. The CT scan showed a tumor mass originating from the sternocleidomastoid muscle, most of which was in the Va and Vb levels of the neck, measuring 40 × 27 × 35 mm, and with a conglomerate of lymph nodes in the IV level measuring 26 × 22 × 23 mm (Figures 2(a)–2(c)).

**Figure 3:**
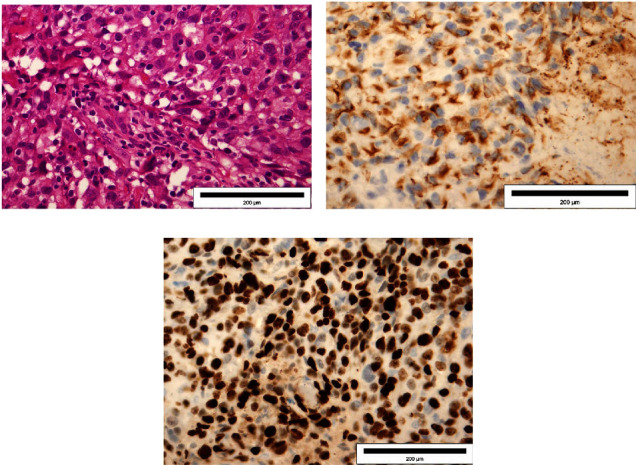
Microscopic appearance of the samples. Hematoxylin & eosin (a). Cell cytoplasm showed dot-like positive staining for CK (b). Ki-67 stained 90% of tumor cells (c). Magnification ×40.

**Figure 4:**
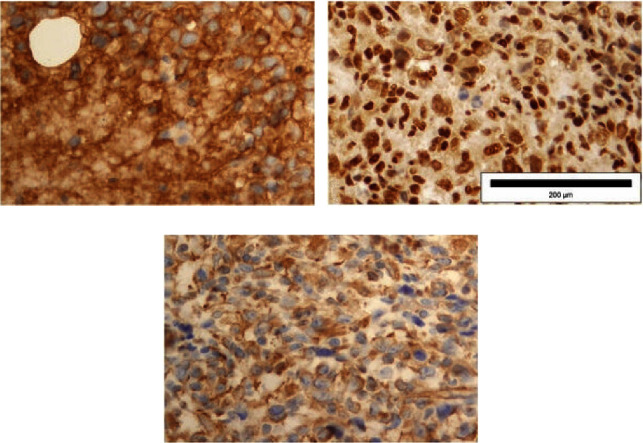
Microscopic appearance of the samples. Tumor cells showed strong and diffuse positive staining for CD99 (a), FLI (b) and vimentin (c). Magnification ×40.

**Figure 5:**
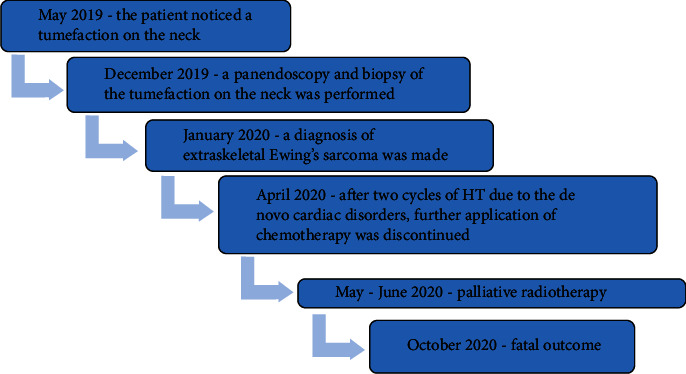
Timeline figure depicting symptom onset, diagnostic workup, therapeutic interventions, complications, disease progression, and death of patients.

Chest radiography and abdominal ultrasound findings was normal. Panendoscopy with blind biopsies were performed, but the pathohistological findings of the samples were negative for malignancy. Afterwards, an open biopsy of the neck mass was performed. Pathohistological and immunohistochemical findings revealed Ewing's sarcoma. The cell cytoplasm showed dot-like positive staining for CK. Ki-67 stained 90% of tumor cells (Figures 3(a)–3(c)). Tumor cells showed strong and diffuse positive staining for vimentin, CD99, and FLI (Figures 4(a)–4(c)). Tumor cells were negative for p16, p63, p40, CK5/6, CK7, S-100, SOX-10, synaptophysin, chromogranin A, CD56, desmin, ASMA, CD34, CD31, and F-VIII. Afterwards, the patient was presented to the Oncology Advisory Board for Sarcomas, where a decision was made to start treatment with neoadjuvant chemotherapy according to the ADM-IFO protocol (Ifosfamide 2000 mg/m^2^ and Adriamycin 30 mg/m^2^). The patient received two cycles of chemotherapy; the period between the two cycles was 21 days. Due to the *de novo* cardiac disorders, further application of chemotherapy was discontinued. After that the Oncology Advisory Board decided to continue treatment with radiotherapy due to the disease progression in the neck. Palliative radiotherapy was performed using the 3D conformal radiation technique with TD 30 Gy in 15 sessions. Following the initial stabilization, the disease progressed. Unfortunately, the patient passed away nine months after the initial diagnosis. The complete course of treatment for the patient from the onset of symptoms to the death outcome is given in Figure 5.

## 3. Discussion

In 1969, Tefft, Vawter, and Mitus were the first to describe four extraosseous soft tissue sarcomas of the paravertebral region [1–3, 5, 6, 9]. EES is a rare, fast-growing malignant tumor manifesting histomorphological similarities to bone ES [7, 8]. It is an unusual variant of soft tissue ES [10]. It most commonly occurs in younger patients aged 5 years and in the 30–50 age group, which is in contrast to ES of the bone which most commonly develops in children [1, 2, 6].

ES rarely occurs in the head and neck region, while EES is even more rare. The EES tends to form in the paravertebral region. Shimada et al. reported 84 cases of EES after a pathological examination within the Intergroup Rhabdomyosarcoma Study (IRS-I and IRS-II). They registered 15 cases with localization in the head and neck (17.8%), which is a slightly higher percentage compared to that reported in other studies (5–11%) [2, 11]. The Euro-EWING 99 Study database, covering the period from 1999 to 2014 registered 1135 patients with ES, of which 5.02% (57 patients) had primary ES of the head and neck. Of these 57 primary tumors, only 8.77% of cases had EES [12].

EES does not have a specific clinical presentation. About two-thirds of patients report a rapidly growing mass that is usually less painful than skeletal [2, 7, 9, 12, 13]. Despite its tendency to spread locally, EES usually has a pseudocapsule, making it well circumscribed on the CT scan or MRI [7, 12, 13]. An open biopsy is always required for a final diagnosis. There are a large number of tumors to be considered in the differential diagnosis [2, 7, 9, 13].

Immunohistochemical and genomic studies are crucial for making an accurate diagnosis [10]. Immunohistochemical staining is usually different and shows diffuse CD99 (MIC2), FLI-1, and vimentin positivity [2, 4, 12]. Identification of the MIC2 gene by staining with the CD99 marker is very suggestive of EES [2, 5–8, 12]. As for our patient, tumor cells showed strong and diffuse positive staining for vimentin, CD99, and FLI.

When it comes to molecular genetic tests, a characteristic chromosomal translocation t (11; 22) (q24; q12) was found in about 85–90% of ES and EES, which can be proven by FISH analysis [1, 4, 5, 7, 11, 13, 14]. Other specific chromosomal translocations are t (22; 21) (q22; q12), then EWS-ETV1 t (7; 22), EWS-ETV4 t (17; 22), and EWS-FEV t (2; 22) [4].

Modern therapy for EES is the same as for all sarcomas from the Ewing family. It consists of chemotherapy for systemic disease control followed by local disease control by extensive surgical resection and/or radiation [10, 13]. The literature reports that surgical resection followed by adjuvant chemotherapy and radiotherapy significantly improves the prognosis of these patients. Several studies have found greater survival rates after wide-margin resections. The integration of ifosfamide and etoposide into therapy has significantly improved response and survival rates [1, 3, 4, 10]. To date, no study has shown clear evidence of increased survival rates with adjuvant radiotherapy [14]. Our patient's treatment started with adjuvant chemotherapy. After two cycles, chemotherapy was discontinued due to cardiac problems, and treatment was continued with palliative radiotherapy. The patient was treated with both chemotherapy and local radiotherapy due to the advanced stage of the disease; however, he eventually succumbed to this very aggressive disease.

Most reports in the literature state that EES has a worse prognosis than skeletal ES [1, 2, 8, 10]. Comparing patients with skeletal and EES, some recent studies have suggested that patients with localized EES have a less favorable prognosis only in the first two years after diagnosis, following which the prognosis is similar [2, 3, 15]. Patients with metastatic or recurrent disease have a worse outcome, and the five-year survival rate remains about 25% [13]. Venkitaraman et al. analyzed 19 EES patients between 1992 and 2003 and found a 3-year disease-free survival rate of 38% and a 5-year disease-free survival rate of 19% [10]. Literature reports that 25–30% of patients initially have distant metastases that are most common in the lungs and bones [1, 6, 9, 14]. The nine-month period between diagnosis and death in our patient indicates a very poor prognosis for these patients. Also, our patient treated distant metastases during therapy, which is another factor in the poor prognosis.

## 4. Conclusion

Although the radiological appearance and clinical manifestation of EES are nonspecific, they should be considered in the differential diagnosis of soft tissue tumor of the neck to make an accurate diagnosis as early as possible. Given the tumor aggressiveness, early diagnosis and intensive therapy are crucial for a good prognosis. The presence of metastases is an important prognostic factor for ESS. The response to chemotherapy remains one of the most important prognostic factors influencing the survival rates of patients with EES, and in this regard, constant efforts should be made to improve chemotherapy protocols.

## Data Availability

The (radiological and pathohistological findings) data used to support the findings of this study are included within the article.
